# Multiscale Shakedown Capacity Prediction of Parameterized Lattices Under Biaxial Loading Using Ensemble Learning

**DOI:** 10.3390/ma19163425

**Published:** 2026-08-12

**Authors:** Lizhe Wang, Hang Yuan, Wenwen Yuan

**Affiliations:** 1 Contemporary Amperex Future Energy Research Institute (Shanghai) Limited, Shanghai 201108, China; wanglz@catl.com; 2 College of Information Engineering, Shanghai Maritime University, Shanghai 201306, China; 3 School of Advanced Technology, Xi’an Jiaotong-Liverpool University, Suzhou 215123, China

**Keywords:** shakedown analysis, ensemble learning, lattice, energy storage container

## Abstract

The structural lightweighting of next-generation containerized energy storage systems requires reliable fatigue design methodologies for architected lattice materials subjected to complex multiaxial service loading. Despite extensive studies on static performance, fatigue capacity prediction of parameterized lattices remains computationally demanding and experimentally fragmented, particularly when cyclic characteristics cannot be idealized as simple periodic histories. This work develops a unified multiscale evaluation platform grounded in shakedown theory to directly predict multiaxial fatigue capacity for lattice structures without explicit cycle counting. A topology-agnostic nodal-coupling periodic boundary formulation ensures consistent homogenized response evaluation across diverse unit-cell geometries. Numerical robustness in stress computation is achieved through full-integration tetrahedral discretization (FITD), enabling stable treatment of bending-dominated lattice members. To facilitate rapid exploration of high-dimensional design spaces, an ensemble-learning surrogate is trained on multiscale shakedown datasets for capacity prediction and parameter sensitivity analysis. The framework is demonstrated on body-centered cubic and peanut-like auxetic lattices relevant to lightweight container structures. Validation studies confirm accurate FITD scheme-based shakedown fatigue loading prediction. The surrogate model achieves high predictive precision, and parametric analysis reveals topology-dependent fatigue drivers, establishing quantitative linkages between mesoscale geometric variables and shakedown-based fatigue capacity. The proposed methodology provides an efficient and scalable route for fatigue-oriented lattice design and optimization in energy storage container applications.

## 1. Introduction

The increasing penetration of intermittent renewable energy into modern power systems has created a growing demand for large-scale energy-storage technologies that can balance power supply and demand and improve grid reliability. Battery energy storage systems (BESSs) have consequently become an important technical solution for renewable-energy integration, while their safety and structural reliability have also received increasing attention [1,2,3]. For large-capacity applications, containerized BESSs integrate battery modules, thermal-management equipment, electrical components, and supporting structures within standardized enclosures, thereby facilitating manufacturing, transportation, installation, and maintenance. However, the container structure must carry substantial equipment mass and withstand complex mechanical loads arising from lifting, road and marine transportation, installation, and long-term service vibration. Reducing the non-active structural mass while maintaining adequate stiffness, strength, and fatigue resistance has therefore become a critical challenge in the development of the next-generation energy storage container (ESC). Architected lattice materials provide a promising route to address this challenge because their effective mechanical properties can be tailored through unit-cell topology and geometric parameters. With recent advances in computational design and manufacturing technologies, lattice structures are increasingly being considered as lightweight alternatives to conventional solid structural components. Their high specific stiffness, strength-to-weight ratio, energy-absorption capacity, and geometrically tunable mechanical response make them particularly attractive for lightweight ESC applications.

Lattice structures can be designed to satisfy the mechanical requirements of specific service conditions. Owing to their high energy absorption, superior strength-to-weight ratio, and strong impact resistance, they have been widely explored in various engineering applications [4,5]. Accordingly, understanding their mechanical behavior under different loading conditions is essential. Existing studies have focused primarily on compressive performance, partly because compression is common in practice and relatively easy to test. These studies have shown that periodic unit-cell architectures lead to mechanical and deformation behaviors markedly different from those of bulk metallic materials. For instance, Zeng et al. [6] reported that the elastic modulus and yield strength of lattice structures are highly sensitive to geometric variations in the unit cell. With advances in experimental techniques, increasing attention has also been paid to tensile behavior. Zhong et al. [7] reviewed the tensile properties of lattices with different topologies and found that tetrahedral lattices exhibit higher ultimate tensile strength than diamond and body-centered cubic (BCC) lattices. In addition, previous studies have shown that smaller cell sizes generally improve structural stiffness, while relative density, which reflects porosity, strongly affects tensile performance. Therefore, porosity-related parameters, such as strut size, wall thickness, and cell size, play coupled roles in governing stiffness and tensile load-carrying capacity.

Nevertheless, compared with the extensive research on compressive and tensile behavior, fatigue performance of lattice structures remains far less explored, both in fatigue life prediction and fatigue damage assessment. One major reason is the lack of standardized fatigue testing methods, which makes specimen and gripping-end design difficult for lattices with diverse topologies [8]. In addition, conventional uniaxial *S-N* or ∆ε*-N* approaches may not adequately characterize structures subjected to multiaxial loading, especially when the cyclic loading path is uncertain or non-proportional [9]. The mismatch between idealized laboratory loading conditions and actual service environments therefore presents a further challenge to reliable fatigue evaluation. To address these limitations, several numerical approaches have been proposed. Refai et al. [10] investigated the effect of cell topology on multiaxial fatigue strength and established a quantitative relationship between porosity and fatigue resistance using the Crossland criterion. Hedayati et al. [11] assessed the influence of porosity on lattice fatigue life using a damage propagation algorithm validated against experiments. More recently, shakedown analysis, also referred to as a direct method, has been applied to estimate the cyclic load-carrying capacity of composite and architected materials without exact histories [12,13,14,15,16]. Elastic shakedown represents an asymptotic response in which an elastoplastic structure ceases to accumulate additional plastic strain after a finite number of loading cycles. This state is closely related to high-cycle fatigue resistance, for which damage is commonly associated with the accumulation of cyclic plastic strain at the micro- or mesoscale [17]. Accordingly, combining shakedown theory with multiscale analysis provides a rational means of estimating the fatigue limit of heterogeneous structures without explicitly tracing the complete loading history [18,19]. In particular, Melan’s lower-bound theorem can be used to identify a conservative load domain within which progressive plastic deformation is prevented, thereby providing an efficient estimate of the structural fatigue limit under cyclic loading with uncertain paths. Within this framework, the shakedown-based fatigue criterion introduced by Dang Van avoids computationally intensive incremental procedures and is well suited to multiaxial loading with non-prescribed cyclic histories [20,21]. Although this approach can be reformulated as a large-scale convex mathematical programming problem and has been further optimized [22,23,24], multiaxial fatigue analysis of lattice structures remains challenging. In ESC applications, these difficulties are amplified by the large structural scale and the disparity between macroscale container dimensions and mesoscale lattice features, which lead to extremely large finite-element (FE) models and high evaluation cost. Consequently, fatigue studies on ESCs remain limited, and investigations of lattice structures integrated into ESCs are even rarer. At present, no general method is available for predicting the multiaxial fatigue capacity of ESC lattice structures under service conditions with unknown cyclic paths, such as random vibrations during road and sea transportation. Moreover, quantitatively linking mesoscale design parameters to structural fatigue behavior remains difficult.

**Figure 1:**
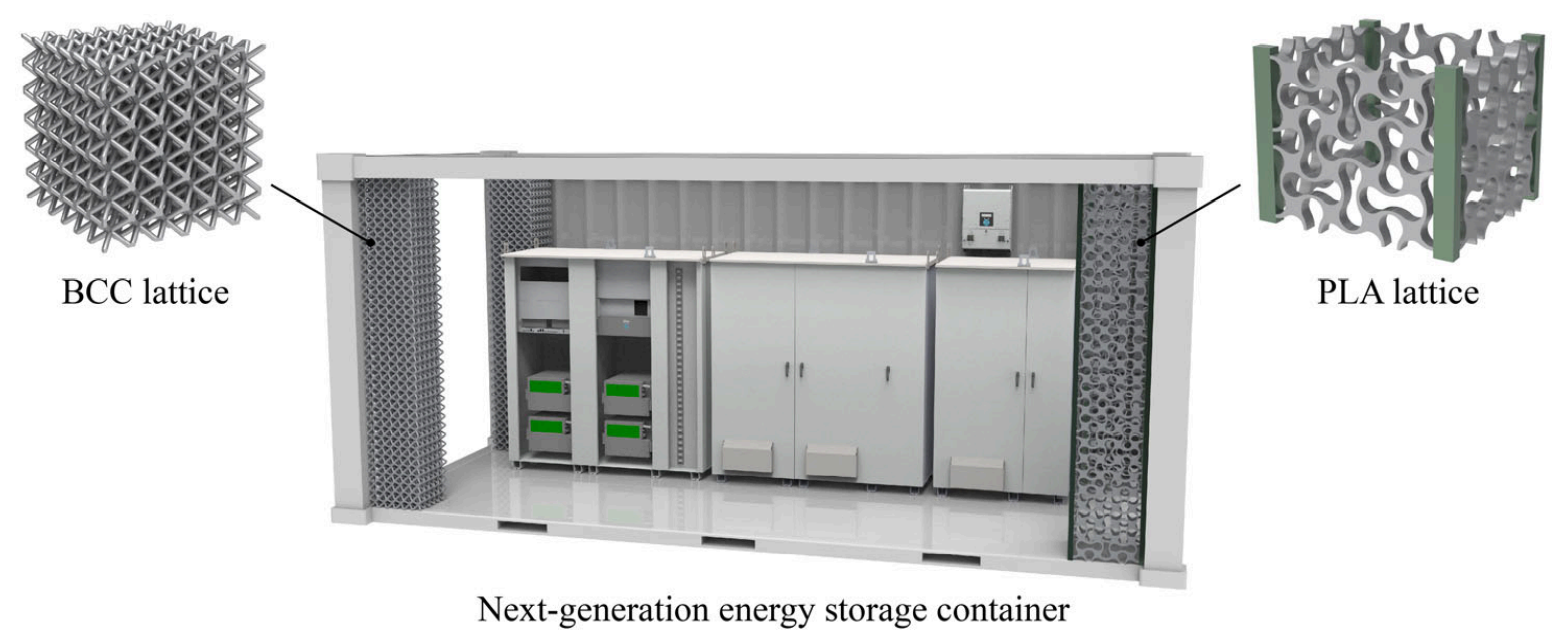
Schematic of lattice-member integration in next-generation ESCs.

To address these limitations, this study develops a unified multiscale framework for predicting the shakedown-based fatigue capacity of parameterized lattice structures intended for next-generation ESCs. As shown in Figure 1, two lattice topologies, BCC and peanut-like auxetic (PLA), are considered. The scientific contributions are threefold. First, a simplified nodal-coupling periodic-boundary formulation is introduced to impose periodic boundary conditions (PBCs) on lattice RVEs with non-matching tetrahedral surface meshes, thereby avoiding direct node-to-node correspondence while preserving the required macroscopic–mesoscopic consistency. Second, a full-integration tetrahedral discretization (FITD) scheme is incorporated into the lower-bound shakedown formulation to improve the evaluation of elastic and residual stress fields in geometrically complex, bending-dominated lattice members. Its accuracy is assessed through benchmark comparisons and cyclic incremental analyses. Third, an ensemble-learning-based machine learning model (ELM) is constructed to establish a nonlinear mapping between mesoscale geometric variables, biaxial loading conditions, and the shakedown factor. Once trained using the high-fidelity shakedown dataset, the surrogate predicts the capacity of new parameter combinations without repeated finite-element analysis and GUROBI-based optimization. The resulting framework therefore provides not only a loading-history-independent fatigue-capacity criterion, but also an efficient means of quantitatively linking lattice geometry to multiaxial structural performance.

## 2. Methodology

### 2.1. Homogenization-Based Periodic Boundary Conditions Applied on Lattice

**Figure 2:**
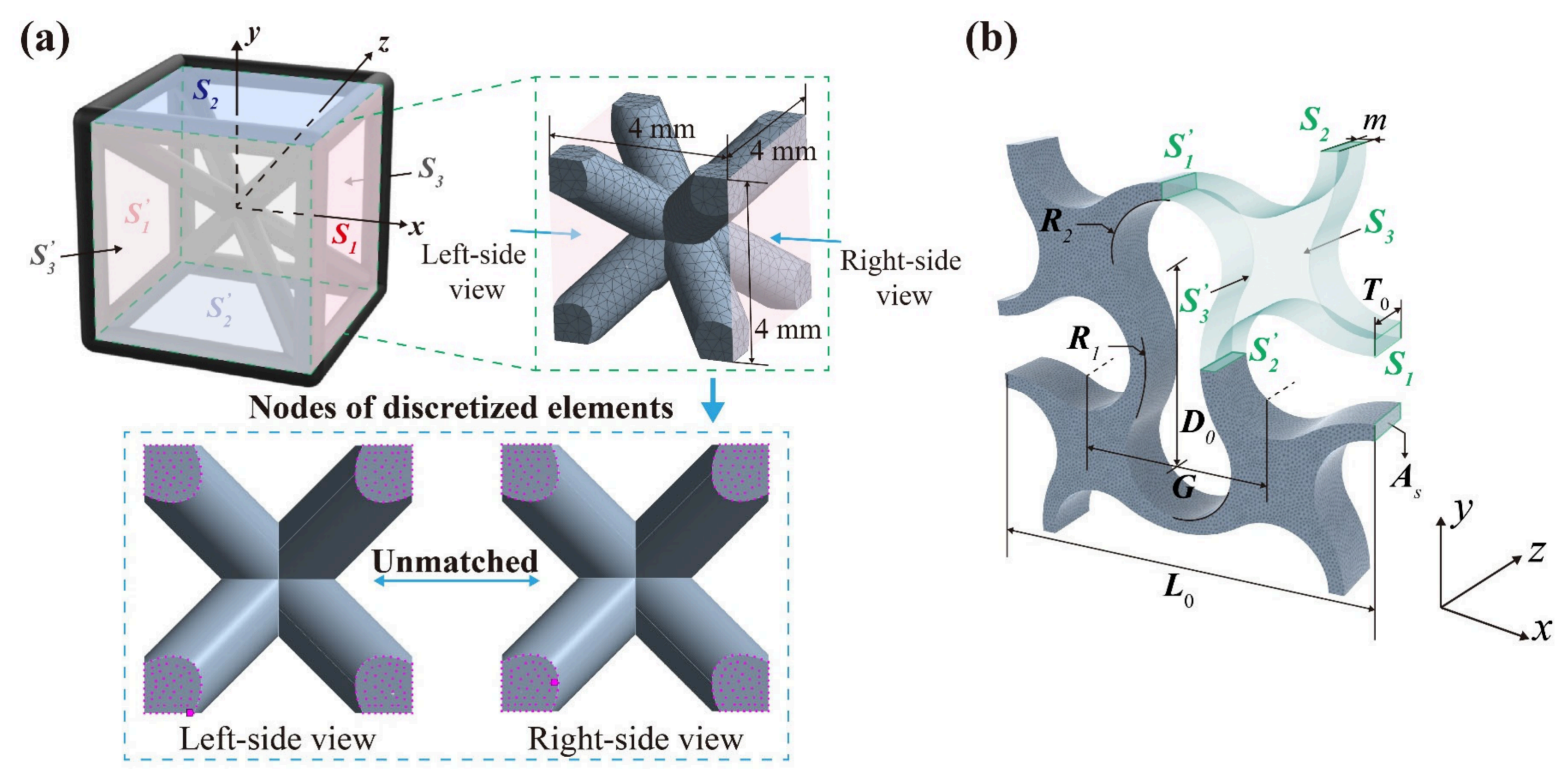
Numerical realization of NCPBCS for parameterized lattice RVES, including (**a**) a BCC+CP lattice with non-matching surface meshes and (**b**) a PLA lattice.

For three-dimensional lattice unit cells discretized using tetrahedral free meshes, the meshes on opposite periodic surfaces are generally non-matching, as illustrated in Figure 2a. This makes the implementation of conventional constraint-equation-based PBCs difficult, since such approaches typically require matching nodes or elements on corresponding periodic surfaces, which is especially challenging for lattice structures [25,26]. As a result, enforcing matched surface meshes is often unavoidable, potentially degrading mesh quality and numerical accuracy.

To overcome this limitation, a simplified nodal-coupling periodic boundary condition formulation (NCPBC) is adopted. Following our previous studies [27,28], the body-centered cubic lattice with cubic-primitive members (BCC+CP) and the PLA lattice considered for applications of ESCs are used to demonstrate its implementation, as shown in Figure 2. For the loading cases examined here, the principal loading directions coincide with the geometric symmetry axes of the unit cell. The deformation and stress fields within the representative volume element (RVE) can therefore satisfy both symmetry and periodicity. On this basis, the periodic response along the three orthogonal directions is imposed through a combination of symmetry constraints and directional coupling conditions. This formulation avoids direct node-to-node matching on opposite surfaces and provides an efficient means of establishing periodic connections for the geometrically distinct lattice RVEs considered in this study.

The lattice RVEs are defined in the Cartesian coordinate system (x,y,z) with unit vectors $\left({\vec{e}}_{x},{\vec{e}}_{y},{\vec{e}}_{z}\right)$, as shown in Figure 2. The considered lattice units exhibit in-plane periodicity, meaning that the surfaces in the x-y plane are periodic counterparts, while the z direction is non-periodic. On this basis, a reliable and computationally efficient evaluation can be performed using only one-quarter of the PLA lattice RVE instead of the full model. In this setting, surfaces $S_{i}^{\prime }\left(i=1,2\right)$ are defined as symmetry planes to ensure that their planar condition is preserved during RVE deformation. For the associated periodic surfaces $S_{i}\left(i=1,2\right)$, only directional couplings are required, avoiding complex node-matching equations over the surface meshes. Moreover, for the non-periodic surfaces $S_{3}$ and $S_{3}^{\prime }$ in the ${\vec{e}}_{z}$ direction, additional normal constraints are imposed. The implementation of NCPBCs for the lattice model is therefore given as follows:

(1) $$\{\begin{array}{c} Symmetry:\;S_{1}^{\prime }:\;u_{x}{\overset{⃑}{e}}_{x}=0;\;S_{2}^{\prime }:\;u_{y}{\overset{⃑}{e}}_{y}=0\;\\ Periodicity:\;S_{1}:\;u_{x}{\overset{⃑}{e}}_{x}=C_{x};\;S_{2}:\;u_{y}{\overset{⃑}{e}}_{y}=C_{y}\\ Normal\;plane:\;S_{3}:\;u_{z}{\overset{⃑}{e}}_{z}=C_{z1};\;S_{3}^{\prime }:\;u_{z}{\overset{⃑}{e}}_{z}=C_{z2} \end{array}$$

where $C_{x}$, $C_{y}$, $C_{z1}$ and $C_{z2}$ denote constant displacements in the x, y, and z directions, respectively, indicating that the cross sections $S_{i}\;(i=1,2,3)$ and $S_{3}^{\prime }$ are coupled in their corresponding directions.

As illustrated in Figure 2, in this study, the BCC lattice has one independent local design variable, the rod diameter d, whereas the PLA lattice has four: the outer radius $R_{1}$, inner radius $R_{2}$, edge width m, and center distance $D_{0}$. The BCC unit cell is defined with dimensions of 4 mm × 4 mm × 4 mm, and the constant thickness $T_{0}$ of the PLA lattice RVE is set to 4 mm. For the PLA lattice, two additional dependent parameters and the cross-sectional area $A_{s}$ are calculated using Equation (2).

(2) $$\{\begin{array}{c} G=2\sqrt{{\left(R_{1}+R_{2}\right)}^{2}-{D_{0}}^{2}/4}\\ L_{0}=D_{0}+2\times R_{2}+G-2\times R_{1}+2\times m\\ A_{s}=T_{0}\times m \end{array}$$

To further assess the proposed shakedown-based fatigue-capacity prediction platform, both non-metallic and metallic lattice materials are considered: a photocurable resin (SH8801, UnionTech, Stuttgart, Germany) and SPA-H steel. Owing to its high specific stiffness and specific shear modulus, the resin is employed for the BCC lattice, whereas the PLA lattice is modeled using SPA-H steel. In addition, a 50 μm-thick copper coating is applied to the BCC lattice to meet the electrical-conductivity requirements of selected structural components in the energy storage container. The ranges of the independent geometric variables and the corresponding material properties of the two lattice configurations are summarized in Table 1 and Table 2. All constituent materials are modeled as elastic–perfectly plastic. The corresponding NCPBCs are imposed on the full BCC RVE and the one-quarter PLA RVE in accordance with Figure 2 and Equation (1). A reference tensile pressure of $P_{0}=1$ MPa is then applied independently to the periodic coupling surfaces $S_{1}$ and $S_{2}$. The two load components are denoted by $P_{S_{1}}$ and $P_{S_{2}}$ respectively, and are used to evaluate the biaxial fatigue-loading capacity. The FE analyses are performed using the Static Structural module in ANSYS Workbench 2024 R2 with the direct equation solver. Consistent with the small-deformation assumption adopted in the shakedown formulation, the large-deflection option is disabled in all FE analyses. Based on the mesh-convergence studies reported in Refs. [27,28], element sizes of 0.2 mm and 0.1 mm are adopted for the BCC and PLA lattice RVEs, respectively.

### 2.2. FITD-Based Multiscale Shakedown Evaluation

In the direct method of shakedown theory, the structure Ω is subjected to NL variable loads $P_{i}\left(x,t\right)\left(i=1,2,\ldots ,NL\right)$, each varying within a prescribed range over time t. It is assumed that the structure satisfies Drucker’s stability postulate, experiences only small deformation, and consists of an elastic–perfectly plastic material, while hardening and damage are neglected. Accordingly, the loading history P(x,t) is represented by time-independent load bases $P_{i}^{0}(x)$ and corresponding load multipliers $\mu_{i}(t)$. The load domain L is therefore defined as the set of all admissible loading states P(x,t), as follows:

(3) $$L=\left\{P\left(x,t\right)|P\left(x,t\right)=\sum_{i=1}^{NL}\;P_{i}\left(x,t\right)=\sum_{i=1}^{NL}\;\mu_{i}\left(t\right)P_{i}^{0}\left(x\right),\mu_{t}^{-}\leq \mu_{i}\left(t\right)\leq \mu_{t}^{+}\right\}.$$

Here, $\mu_{t}^{-}$ and an $\mu_{t}^{+}$ denote the lower and upper bounds of the load multiplier $\mu_{i}\left(t\right)$, respectively.

Melan’s lower-bound shakedown theorem for elastic–perfectly plastic materials can be stated as follows [29,30]: Suppose that there exist a time-independent self-equilibrated residual stress field $\bar{\rho }\left(x\right)$ and a shakedown load factor $\alpha^{SD}>1$. Then, for any loading path including all vertices ${\hat{P}}_{i}\left(i=1,2,\ldots ,2^{NL}\right)$ of L, the superposition of $\bar{\rho }\left(x\right)$ and the corresponding elastic stress field $\sigma^{E}(x,{\hat{P}}_{i})$ satisfies the yield criterion F(σ) throughout Ω:

(4) $$F\left(\alpha^{SD}\sigma^{E}\left(x,{\hat{P}}_{i}\right)+\bar{\rho }\left(x\right)\right)<0,i=1,2,\ldots ,2^{NL}.$$

Here, the shakedown analysis is conducted using the von Mises yield criterion F(σ). Two independent loads, associated with the load multipliers $\mu_{1}(t)$ and $\mu_{2}(t)$, are considered. For convenience in representing L and characterizing the interaction between the two load components, the magnitudes of the load multipliers are defined as follows:

(5) $$\mu_{1}=cos\theta ,\mu_{2}=sin\theta ,\theta \in \left[0,2\pi \right].$$

Thus, the four vertices of L can be uniformly represented as follows:

(6) $${\hat{P}}_{i}=cos\theta P_{0}+sin\theta P_{0}.$$

Based on the principle of virtual work, the residual stress field can be defined as follows:

(7) $$\int_{\Omega }\;{\left\{\varepsilon^{*}\right\}}^{T}\left\{\bar{\rho }\right\}d\Omega =0.$$

Any arbitrary shape in a global coordinate system (GCS) can be represented discretely using regular-shaped isoparametric elements (i.e., tetrahedral or hexahedral elements). In this context, the virtual strain $\left\{\varepsilon^{*}\right\}$ can be defined as:

(8) $$\left\{\varepsilon^{*}\right\}=B{\left\{\delta \right\}}^{e},$$

where ${\left\{\delta \right\}}^{e}$ represents the nodal displacements of the body structure, and the element geometric matrix B can be defined as $B=\left[\partial \right]{\left[N\right]}_{x,y,z}$ with [∂] representing the derivative operator matrix and ${\left[N\right]}_{x,y,z}$ indicating the shape function of the elements applied for discretization from the perspective of a GCS. Then, applying isoparametric elements in a natural coordinate system (NCS) to represent an arbitrary shape in a GCS can be conducted using the Jacobian Matrix J. For finite elements adopting FITD in this work, ${\left[N\right]}_{x,y,z}$ is expressed as ${\left[N\right]}_{s,t,r}$ according to the orthogonal axis directions *s*, *t*, and *r* of the NCS as follows:

(9) $$N_{1}=s;\;N_{2}=t;\;N_{3}=r;\;N_{4}=1-s-t-r.$$

Introducing $B^{*}$ according to the NCS and integration weight factors w according to the FITD. Then, substituting Equation (8) into Equation (7), replacing B with $B^{*}$, and transforming the volume Ω into an element volume $\Omega_{e}$ according to the number of elements NE reformulates Equation (7) as follows.

(10) $$\int_{\Omega }\;B^{T}\left\{\bar{\rho }\right\}d\Omega =\sum_{e=1}^{NE}\;\left[\int_{\Omega_{e}}\;B^{T}{\left\{\bar{\rho }\right\}}^{e}d\Omega \right]=\sum_{e=1}^{NE}\;\sum_{i=1}^{NGE}\det(J){B^{*}}_{i}^{T}{\left\{{\bar{\rho }}_{i}\right\}}^{e}w_{i}=0$$

where NGE is Gauss point numbers per element. For FITD employed herein, NGE=4 and w=1/24. Equation (10) can be further simplified by defining an element equilibrium matrix $C_{i}^{e}=\det(J){B^{*}}_{i}^{T}w_{i}$, which is also constant. Substituting this expression into Equation (10) yields the following:

(11) $$\int_{\Omega }\;B^{T}\left\{\bar{\rho }\right\}d\Omega =\sum_{i=1}^{NG}\;C_{i}^{e}{\left\{{\bar{\rho }}_{i}\right\}}^{e}=0,$$

where NG=NE×NGE describes the total Gauss point numbers within Ω.

For lattice RVE models with the proposed NCPBCs, the macroscopic pressure loads, equivalent to the applied stress tensor Σ, are imposed on the boundary by assuming that the RVEs are connected in series and subjected to a uniform macroscopic stress state, i.e., $\langle \sigma^{E}\rangle =\Sigma$. Moreover, since the loading directions coincide with the symmetry axes, the displacement fluctuation on the periodic boundaries vanishes, i.e., $\tilde{u}(x)=0$, and the corresponding volume-averaged strain is given by ⟨ε(x)⟩=E. On this basis, the NCPBC-based multiscale shakedown formulation is established by Equation (12) using the full RVE for the BCC lattice and the quarter-RVE for the PLA lattice.

(12) $$\{\begin{array}{c} \begin{array}{c} max:\;\alpha^{SD}\\ F\left(\alpha^{SD}\sigma_{i}^{E}\left({\hat{P}}_{j}\right)+{\bar{\rho }}_{i}\;\left(x\right)\right)<0,\;\forall i\in \left[1,NG\right],\;\forall \;j\in \left[1,4\right]\\ \left[C\right]\left\{\bar{\rho }\right\}=\det\left(J\right)B^{*T}\left\{\bar{\rho }\right\}=0\\ \mathrm{div}\;\left(\sigma^{E}\right)=0\;\;\;\;\;\;\;\;\;\;\;\;\;\;\;\;in\;\Omega_{\mathrm{RVE}}\\ \left\langle \varepsilon \right\rangle =E\\ \sigma^{E}=D:\varepsilon =D:E\;\;\;\;\;\;\;\;\;\;in\;\Omega_{\mathrm{RVE}}\\ \left\langle \sigma^{E}\right\rangle =\Sigma \\ u^{s}\;\;\;\;\;\;\;\;\;\;\;\;\;\mathrm{symmetric}\;on\;{\partial \Omega }_{\mathrm{RVE}} \end{array}\\ \tilde{u}=\tilde{\varepsilon }=0\;\;\;\;periodic\;on\;{\partial \Omega }_{\mathrm{RVE}}\\ \sigma^{E}\cdot n\;\;\;\;\;anti\text{-}periodic\;on\;{\partial \Omega }_{\mathrm{RVE}} \end{array}.$$

The final optimization problem is solved using an interior-point algorithm implemented in the GUROBI solver.

### 2.3. Ensemble Learning-Based Predictive Model

**Figure 3:**
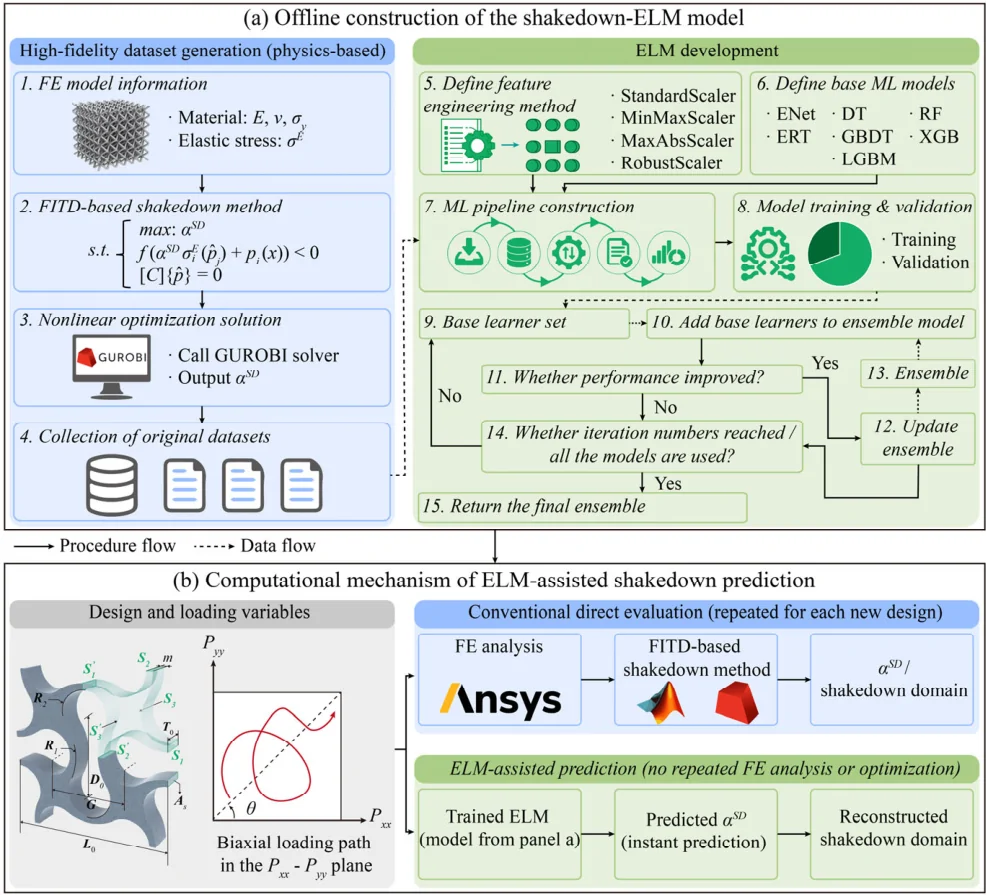
Integrating ELM with shakedown analysis: (**a**) flowchart of shakedown load-factor evaluation for parameterized lattices using the ELM platform; (**b**) computational advantage of ELM-assisted prediction compared with conventional direct evaluation.

The computational burden of solving the large-scale shakedown optimization problem in Equation (12) increases substantially with the number of finite elements required to resolve the mesoscale geometry of lattice structures. This motivates the development of an efficient data-driven surrogate for exploring large parameter spaces. As illustrated in Figure 3b, two routes can be used to evaluate the shakedown capacity of previously unseen combinations of geometric and loading parameters. In the conventional route, the FE model must be analyzed and the corresponding mathematical optimization problem solved separately for each new parameter combination, providing high-fidelity results at considerable computational cost. By contrast, once trained using the shakedown dataset generated during the offline stage, the ELM establishes a direct mapping from the input variables to the shakedown factor and the associated capacity domain. Consequently, predictions for new designs can be obtained without repeatedly performing FE analyses and GUROBI-based optimization. The proposed approach therefore enables more efficient design-space exploration and facilitates quantitative assessment of the effects of geometric and loading parameters on lattice shakedown capacity.

Prediction of the shakedown loading-capacity domain of parameterized lattice structures is essentially a regression problem. In the proposed machine learning framework, the input layer contains the independent design variables and the load variable θ in Equation (6), which denotes the proportion of biaxial loads in two orthogonal directions, while the output layer represents the shakedown load factor $\alpha^{SD}$ and the corresponding shakedown fatigue domain. To capture the relationship between these variables, an ELM composed of multiple base learners was constructed. Following an ensemble-learning framework previously applied to fatigue-life prediction of a pump-spindle assembly with parameterized geometry [31], the candidate pool considered in this study comprises Elastic Net (ENet), decision tree (DT), Random Forest (RF), Extremely Randomized Trees (ERT), Gradient Boosted Decision Trees (GBDT), XGBoost (XGB), and Light Gradient Boosting Machine (LGBM). The ensemble-learning framework is implemented in Python 3.13.5. Data preprocessing and the ENet, DT, RF, ERT, and GBDT models are implemented using scikit-learn, whereas XGB and LGBM are implemented using the XGBoost and LightGBM libraries, respectively. The forward stepwise and Caruana ensemble-selection procedures are implemented through custom Python 3.13.5 scripts. Forward stepwise selection was first applied to sequentially add models and optimize ensemble performance. However, because this procedure may overfit the validation set, Caruana ensemble selection was further adopted, incorporating selection with replacement, sorted ensemble initialization, and bagged ensemble selection [32]. As a result, the final fusion model can be constructed with improved robustness and reduced risk of overfitting.

The hyperparameters of the candidate pipelines were assessed using five repeated random holdout evaluations, and the settings producing the lowest mean NRMSE were retained. For the MinMaxScaler–RF pipeline, the number of trees, maximum tree depth, maximum number of features considered at each split, minimum number of samples required to split an internal node, and minimum number of samples per leaf were set to 400, 12, 1.0, 4, and 1, respectively. For the RobustScaler–ENet pipeline, the regularization coefficient α and L1 mixing ratio were set to $3.0\times 10^{-5}$ and 0.95, respectively, with a maximum of 20,000 iterations and a convergence tolerance of $1.0\times 10^{-4}$. For the MaxAbsScaler–LGBM pipeline, the number of boosting iterations, learning rate, number of leaves, maximum tree depth, and minimum number of child samples were set to 500, 0.02, 16, 7, and 25, respectively. The subsampling ratio, feature-sampling ratio, L1 regularization coefficient, and L2 regularization coefficient were set to 0.75, 0.85, 0.01, and 0.10, respectively. Hyperparameters not specified above were retained at their default values.

To evaluate the predictive performance of the ELM, several performance metrics are employed. Among them, the normalized root mean squared error (NRMSE) enables comparison across variables with different scales by normalizing the root mean squared error (RMSE) with respect to the range of the observed data. In this way, NRMSE measures the typical prediction error as a fraction of the total variation in the target variable:

(13) $$NRMSE=\frac{\sqrt{MSE}}{\bar{O}},$$

where MSE is the expected squared loss or error and expressed as: $MSE\left(y,\hat{y}\right)=\frac{1}{n_{samples}}\sum_{i=0}^{n_{samples}-1}{\left(y_{i}-{\hat{y}}_{i}\right)}^{2}$. Here, $\bar{O}$ signifies the average true value. In addition, the $R^{2}$ score, or the coefficient of determination, gives a measure of the model’s goodness of fit:

(14) $$R^{2}\left(y,\hat{y}\right)=1-\frac{\sum_{i=1}^{n}\;{\left(y_{i}-\hat{y_{i}}\right)}^{2}}{\sum_{i=1}^{n}{\left(y-\bar{y_{i}}\right)}^{2}},\;$$

where $\bar{y}$ represents the mean of the true values, calculated as $\bar{y}=\frac{1}{n}\sum_{i=1}^{n}y_{i}$.

In machine learning, the prediction of numerical responses from a set of input features is generally formulated as a regression problem. To improve model performance, StandardScaler, MinMaxScaler, MaxAbsScaler, and RobustScaler [33] were considered for feature scaling. The predictive performance was assessed using five repeated random holdout evaluations. In each repetition, the complete dataset was randomly partitioned into 70% training samples and 30% testing samples. The model was fitted using the training subset and evaluated on the unseen testing subset, and the reported performance metrics are averaged over the five repetitions. The $R^{2}$ and NRMSE values were calculated separately for the training and testing subsets in each repetition, and the corresponding mean values and standard deviations over the five repetitions were used to assess predictive accuracy and the generalization gap. This procedure reduces the sensitivity of the evaluation results to a particular random partition. The overall workflow of the proposed machine-learning model for lattice shakedown-capacity evaluation is illustrated in Figure 3a.

According to Equation (12), the multiscale shakedown loading capacities of the BCC and PLA lattices are determined using the FITD scheme. The L and the corresponding biaxial tensile pressure components are defined as follows:

(15) $$\{\begin{array}{c} 0\leq P_{S_{2}}\leq \mu_{1}^{+}P_{o}=cos\theta P_{o}\\ 0\leq P_{S_{1}}\leq \mu_{2}^{+}P_{o}=sin\theta P_{o} \end{array}\;\;\;\;\theta \in \left[0,\frac{\pi }{2}\right].$$

For each geometric configuration and loading condition, the $\alpha^{SD}$ is computed using MATLAB 2024b in conjunction with the GUROBI solver. The shakedown fatigue domain is then constructed by connecting the capacity points (associated with $\alpha^{SD}$) obtained at different values of θ. The calculated $\alpha^{SD}$ values are normalized with respect to $P_{0}/\sigma_{y}$, and datasets are generated for both lattice configurations over the prescribed geometric and loading ranges.

The dataset of BCC and PLA lattices used for the seven-quantity regression model was generated from 216 distinct geometric configurations within the parameter ranges specified in Table 1 and Table 2. To improve the generalization capability of the ELM, each configuration was evaluated at 11 prescribed biaxial loading angles, namely $\theta =0^{\circ }$, $10^{\circ }$, $20^{\circ }$, $30^{\circ }$, $40^{\circ }$, $45^{\circ }$, $50^{\circ }$, $60^{\circ }$, $70^{\circ }$, $80^{\circ }$, and $90^{\circ }$. Each geometry–loading combination produced one normalized shakedown factor, $\alpha^{SD}$, yielding a total of 216×11=2376 samples. Each sample contained seven recorded quantities, namely d, $R_{1}$, $R_{2}$, $D_{0}$, m, θ, and $\alpha^{SD}$, with $\alpha^{SD}$ taken as the target response. Of these, 1661 samples obtained from 151 geometric configurations were used for model development, while 715 samples from the remaining 65 configurations were reserved for validation. The mean and sample standard deviation of $\alpha^{SD}$ were 0.2331 and 0.0940 for the complete dataset, respectively. The corresponding mean ± standard deviation values were 0.2290±0.0922 for the model-development dataset and 0.2553±0.1004 for the validation dataset. No additional data-augmentation procedure was applied.

## 3. Numerical Realization and Validation

### 3.1. The Classic Plate-with-a-Central-Hole Model for Validation

**Figure 4:**
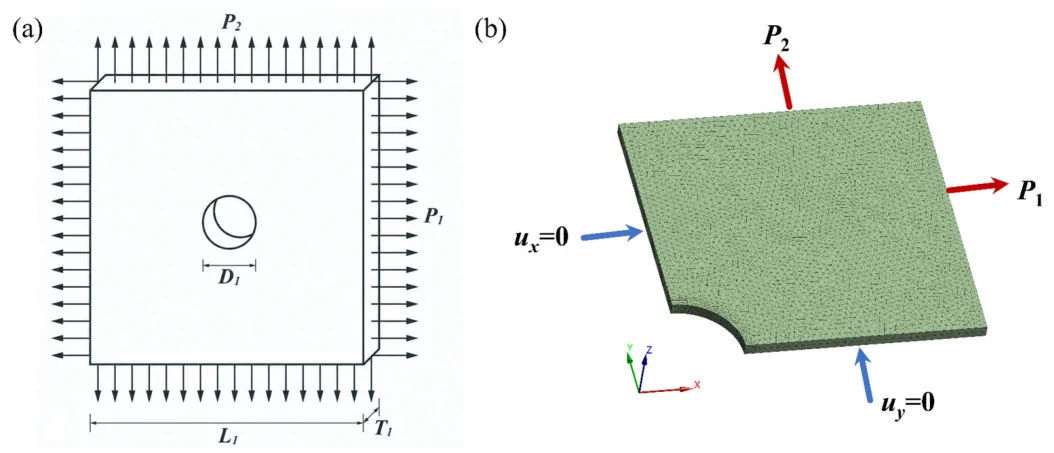
Plate-with-a-central-hole benchmark under biaxial loading: (**a**) schematic graph of the model, (**b**) one-quarter symmetric model discretized using the FITD scheme.

The accuracy of the FITD-based shakedown analysis is evaluated using the classical plate-with-a-central-hole benchmark [34], as illustrated in Figure 4a. The FE geometry is created in ANSYS SpaceClaim 2024 R2. The plate edge length $L_{1}$, thickness $T_{1}$, and hole diameter $D_{1}$ are set to 100 mm, 2 mm, and 20 mm, respectively. Two independent biaxial tensile loads, $P_{1}$ and $P_{2}$, are applied to the corresponding side surfaces and allowed to vary independently up to 100 MPa. According to shakedown theory, the elastoplastic shakedown limit can be determined from the elastic stress field and the material yield strength without explicitly prescribing the complete loading history [22,29]. Therefore, only the material parameters required by the elastic–perfectly plastic constitutive model are specified, as summarized in Table 3. Owing to geometric and loading symmetry, a one-quarter plate model is employed and discretized using the proposed FITD scheme, as shown in Figure 4b. Local mesh refinement is introduced around the hole boundary to accurately capture the associated stress concentration.

To minimize the influence of mesh size on the numerical results, a mesh-convergence study is conducted using the one-quarter symmetric plate model discretized with the FITD scheme. As summarized in Table 4, the maximum and volume-averaged equivalent stresses under $P_{1}=100\;MPa$ of 100 MPa approach stable values once the number of elements exceeds 46,802, with variations remaining within 0.42%. Accordingly, an element size of 1 mm is adopted for the FITD-based plate model as a suitable compromise between numerical accuracy and computational cost.

**Figure 5:**
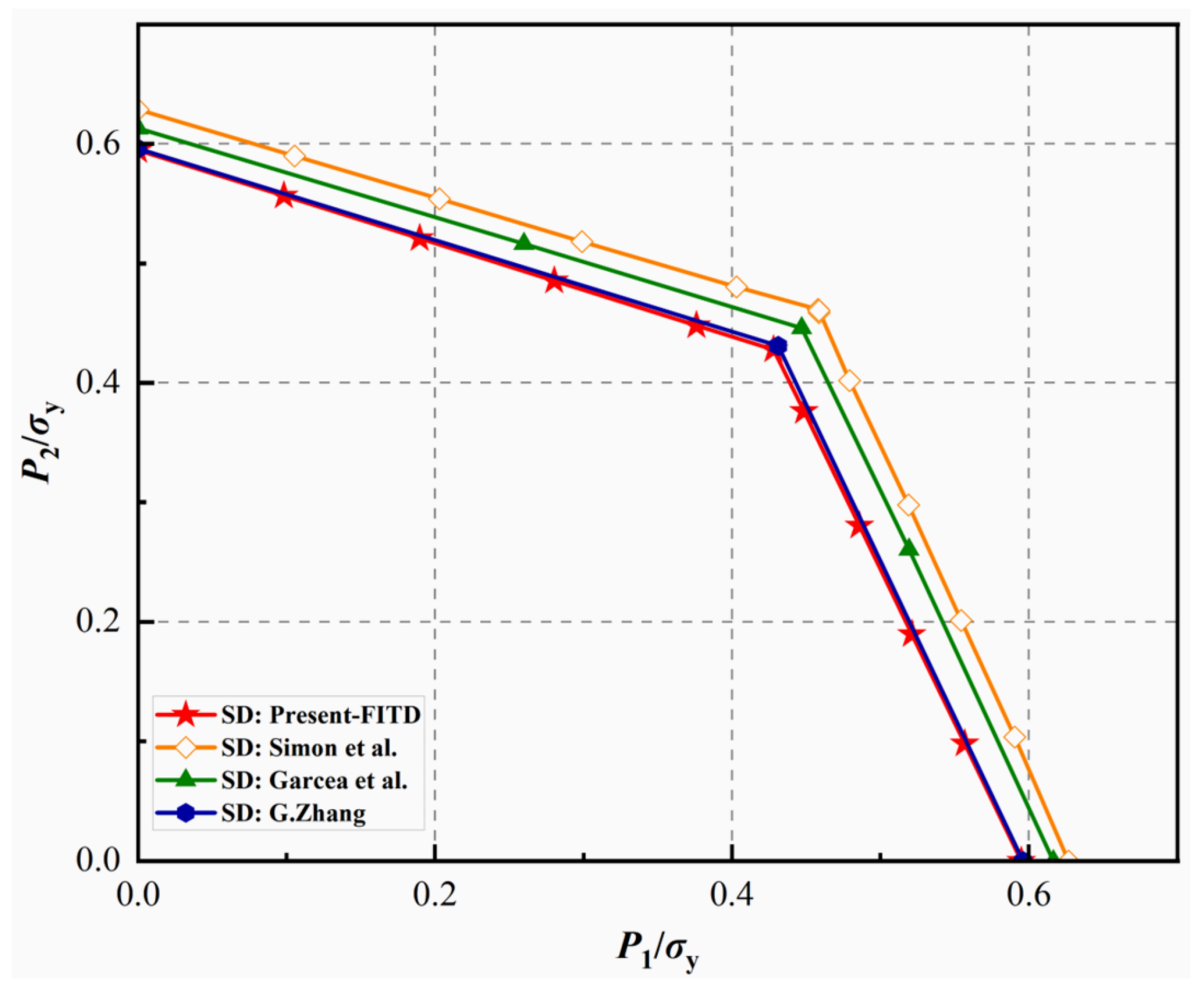
Comparison of shakedown domains obtained by the reference solutions (Simon et al. [34], Garcea et al. [35], G. Zhang [37]) and FITD-based method (SD: the shakedown load domain).

The shakedown load factors are subsequently calculated using the FITD scheme and normalized by $P_{0}/\sigma_{y}$. The resulting values are compared with published reference solutions [34,35,36,37], as summarized in Table 5. Taking the results reported by Zhang [37] as the benchmark, the maximum relative error of the present predictions is 0.7%. The corresponding shakedown domains are then constructed by varying the load ratio $\mu_{1}/\mu_{2}$, and close agreement is obtained with the reference results, as illustrated in Figure 5. These comparisons confirm the accuracy of the FITD-based lower-bound shakedown formulation and support its use in the subsequent analysis of lattice structures.

### 3.2. Validation of NCPBCs-Based Multiscale Analysis on Lattices

**Figure 6:**
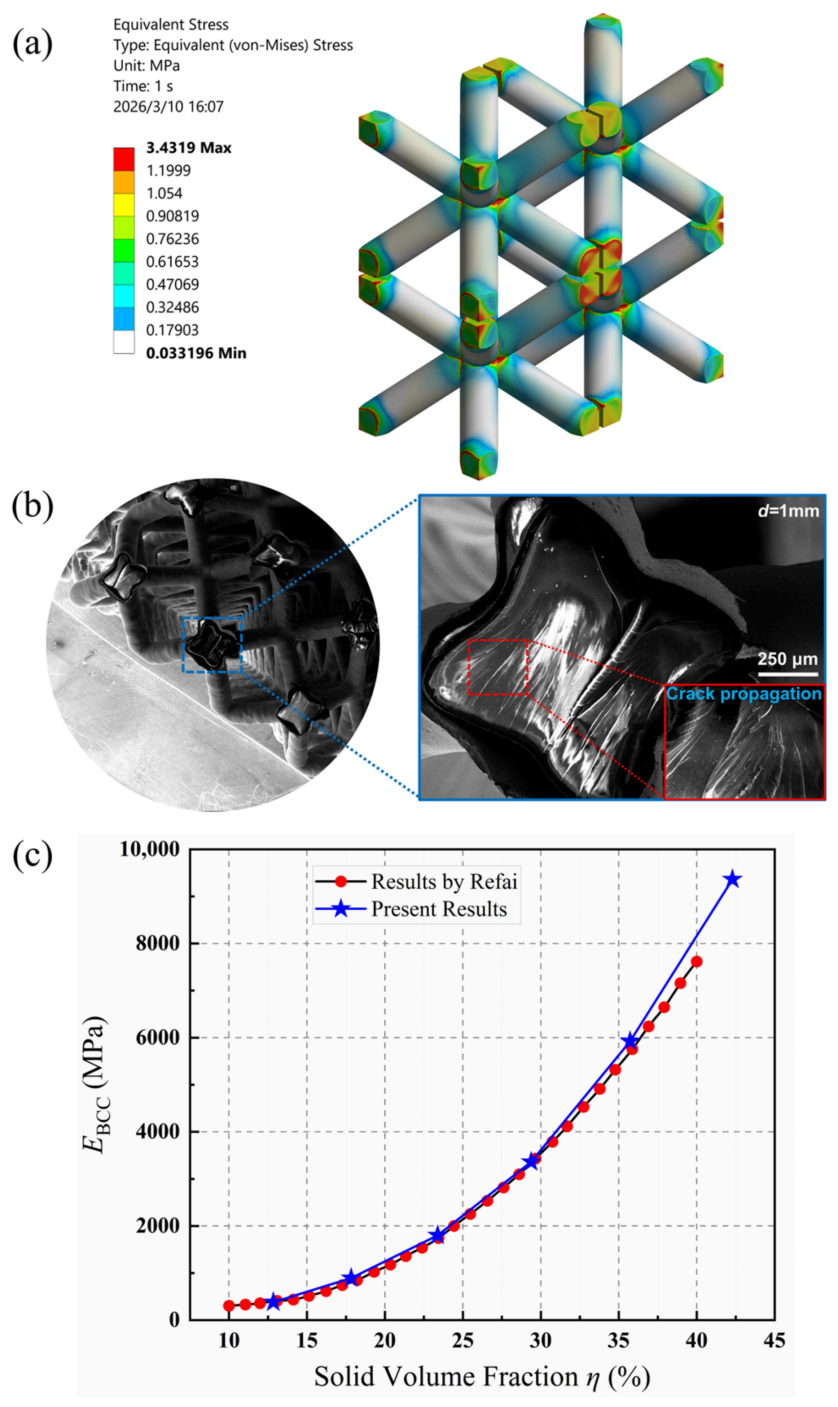
Validation of NCPBCs using the BCC lattice: (**a**) RVE-based lattice contour results of von Mises stress on a global scale; (**b**) SEM image of the cross-section configuration of the BCC lattice structure with d=1 mm [28]; and (**c**) comparison of $E_{BCC}$ based on the RVE analysis with results obtained by Refai [38].

**Figure 7:**
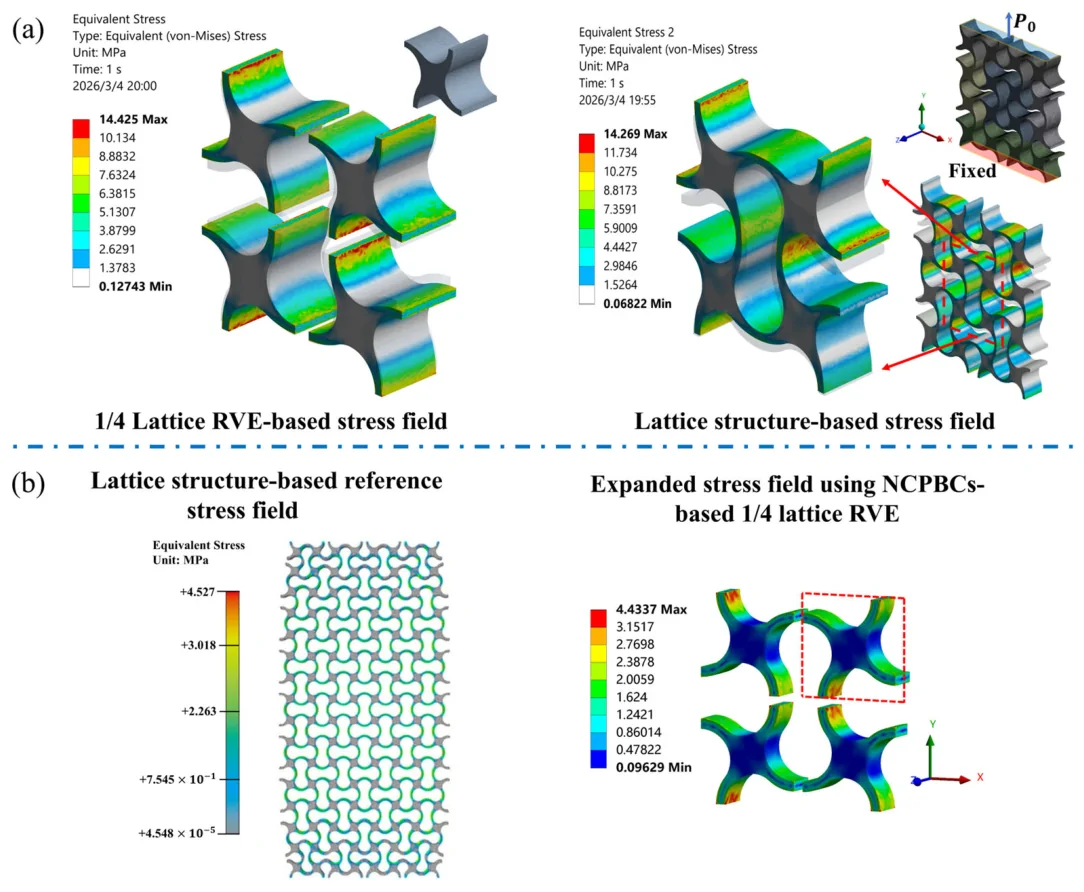
Numerical validation of the NCPBC formulation: (**a**) comparison of the stress fields obtained from the one-quarter RVE and the corresponding full-structure model of the PLA lattice; (**b**) comparison between the stress field predicted by the NCPBC-based RVE model Ref. [27] and the reference full-structure result reported in Ref. [39].

The proposed NCPBCs are validated using lattice structures with distinct geometries, demonstrating their general applicability. For periodic structures under uniform loading, the stress field should also be periodic, provided that appropriate PBCs are imposed on the microscopic model. On this basis, the feasibility and accuracy of the NCPBCs are assessed in two ways. For the PLA lattice, the stress field obtained from the RVE model is compared with that of the corresponding global structure. For the BCC lattice, the stress field and equivalent Young’s modulus $E_{BCC}$ are used for verification through simulation–experiment comparison and comparison with published numerical results, respectively. As shown in Figure 6a, the stress contours predicted by the RVE model are consistent with those of the corresponding structural-scale model, while the locations of peak stress agree with the crack-initiation regions identified in the SEM image in Figure 6b. In addition, the predicted variation of $E_{BCC}$ with rod diameter d, shown in Figure 6c, agrees well with the results reported in the literature [38]. For the PLA lattice, Figure 7a compares the stress fields from the quarter-RVE and global models. To reduce boundary effects, the stress field of the central RVE in the global model is taken as the reference. Only minor differences are observed between the two results. The NCPBC formulation is further assessed using a larger-scale reference lattice structure, for which the detailed geometry, material properties, and loading conditions are available in Ref. [39]. As shown in Figure 7b, the stress contours obtained from the NCPBC-based RVE model closely reproduce those of the full structure. These results indicate that the proposed formulation can consistently relate the macroscopic stress Σ of the lattice structure to the local mesoscale counterpart $\sigma^{E}$ within the RVE. Overall, the comparisons support the accuracy of the NCPBC formulation for the BCC and PLA lattices considered in this study.

### 3.3. Validation of the Loading-History-Independent FITD-Based Shakedown Analysis

This section evaluates the validity and accuracy of extending the direct shakedown method to predict the elastic shakedown capacity of lattice structures without prescribing a specific loading history. The results are verified against those obtained using the conventional incremental method, in which the elastoplastic response is traced step by step over successive loading cycles [40,41,42].

**Figure 8:**
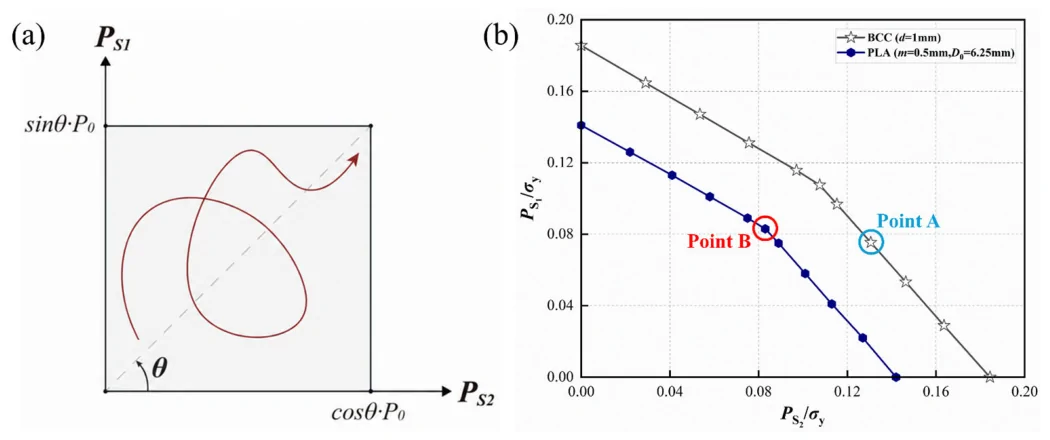
Shakedown evaluation of the BCC and PLA lattices: (**a**) L defined by the independently varying biaxial load components $P_{S_{1}}$ and $P_{S_{2}}$, together with θ; (**b**) shakedown fatigue factor domain of the lattices with the baseline geometric parameters (Point A: biaxial loads at *θ* = 30° for the BCC lattice, Point B: biaxial loads at *θ* = 45° for the PLA lattice).

Based on the L defined in Equation (15) using the biaxial load components $P_{S_{1}}$ and $P_{S_{2}}$ as illustrated in Figure 8a, the shakedown load domains of the BCC and PLA lattices with the baseline geometric parameters listed in Table 1 and Table 2 are constructed, as shown in Figure 8b. Two representative boundary points are selected for the verification: point A at *θ* = 30° for the BCC lattice and point B at *θ* = 45° for the PLA lattice. The corresponding loading domains, denoted as Domain A and Domain B, are defined using the calculated limiting load components $P_{S_{1}}^{*}$ and $P_{S_{2}}^{*}$ as follows:

$$\mathrm{Domain}\;A:\;\{\begin{array}{c} P_{S_{1}}^{*}=\alpha_{A}^{SD}\cdot \sigma_{y}\cdot \sin30{}^{\circ}=0.152\cdot 80\cdot \frac{1}{2}=6.1\;\mathrm{MPa}\\ P_{S_{2}}^{*}=\alpha_{A}^{SD}\cdot \sigma_{y}\cdot \cos30{}^{\circ}=0.152\cdot 80\cdot \frac{\sqrt{3}}{2}=8.7\;\mathrm{MPa} \end{array}$$

$$\mathrm{Domain}\;B:\;\{\begin{array}{c} P_{S_{1}}^{*}=\alpha_{B}^{SD}\cdot \sigma_{y}\cdot \sin45{}^{\circ}=0.117\cdot 355\cdot \frac{\sqrt{2}}{2}=29.4\;\mathrm{MPa}\\ P_{S_{2}}^{*}=\alpha_{B}^{SD}\cdot \sigma_{y}\cdot \cos45{}^{\circ}=0.117\cdot 355\cdot \frac{\sqrt{2}}{2}=29.4\;\mathrm{MPa} \end{array}$$

**Figure 9:**
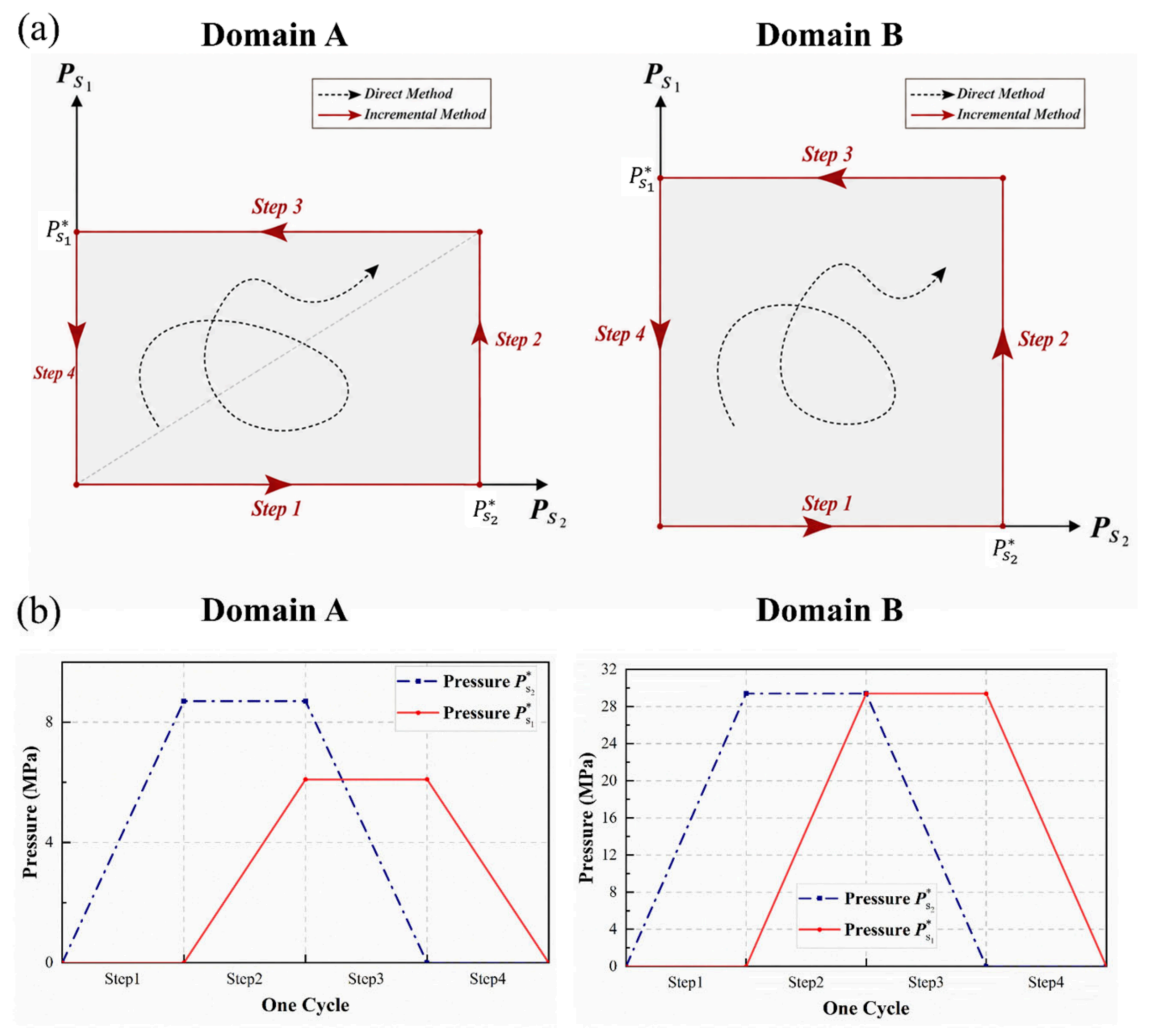
Prescribed cyclic loading paths used in the incremental analyses: (**a**) load-step sequences defined within Domains A and B; (**b**) variations of the biaxial stress components over one loading cycle for Domains A and B.

**Figure 10:**
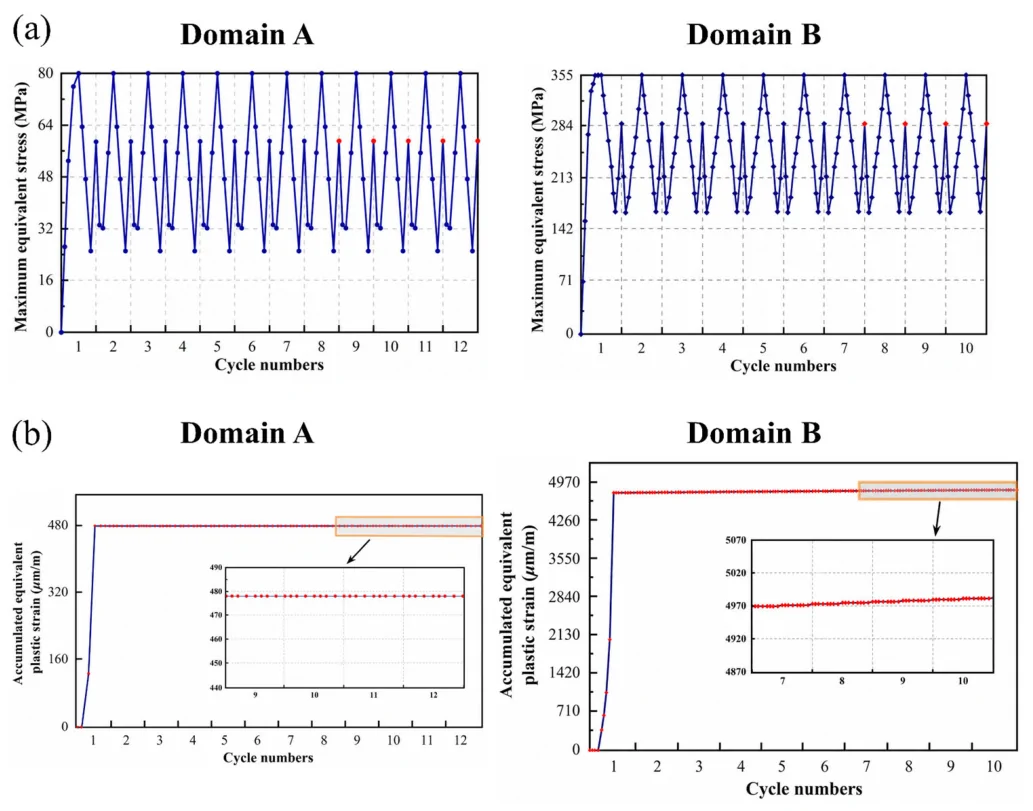
Incremental-analysis results for the BCC and PLA lattices subjected to the prescribed cyclic loading paths in Domains A and B: (**a**) evolution of the maximum von Mises stress over successive cycles; (**b**) convergence of the accumulated equivalent plastic strain with increasing cycle number.

For the incremental analyses, explicit cyclic loading paths are prescribed within Domains A and B, as shown in Figure 9a. The corresponding variations of the two load components over one representative cycle are illustrated schematically in Figure 9b. Twelve loading cycles are applied for Domain A, while ten cycles are applied for Domain B. The cyclic incremental analyses are performed using the Static Structural module in ANSYS Workbench 2024 R2 with the direct equation solver, and the large-deflection option is also disabled. The analyses are conducted using the BCC and one-quarter PLA RVE models with the baseline geometric parameters. The resulting variations in the maximum von Mises stress and accumulated equivalent plastic strain over successive cycles are presented in Figure 10.

Under the elastic–perfectly plastic constitutive assumption, the von Mises stress is bounded by the material yield strength, while a self-equilibrated residual stress field may develop during the cyclic response. In the incremental analyses, the residual stress field is extracted after completion of each prescribed loading cycle, when the applied biaxial loads return to the corresponding reference state. As shown by the red markers in Figure 10a, the residual stress approaches a stable value after several cycles. Meanwhile, the accumulated equivalent plastic strain also converges, with variations below 0.44%, as shown in Figure 10b. These results indicate that both lattice models reach an elastic shakedown state under the prescribed cyclic loading paths. The close correspondence between the incremental responses and the direct shakedown predictions supports the capability of the FITD-based formulation to determine the shakedown capacity without explicitly tracing the full loading history.

## 4. EML-Based Shakedown Loading Capacity Predictions for Lattices

### 4.1. Performance of the ELM

**Figure 11:**
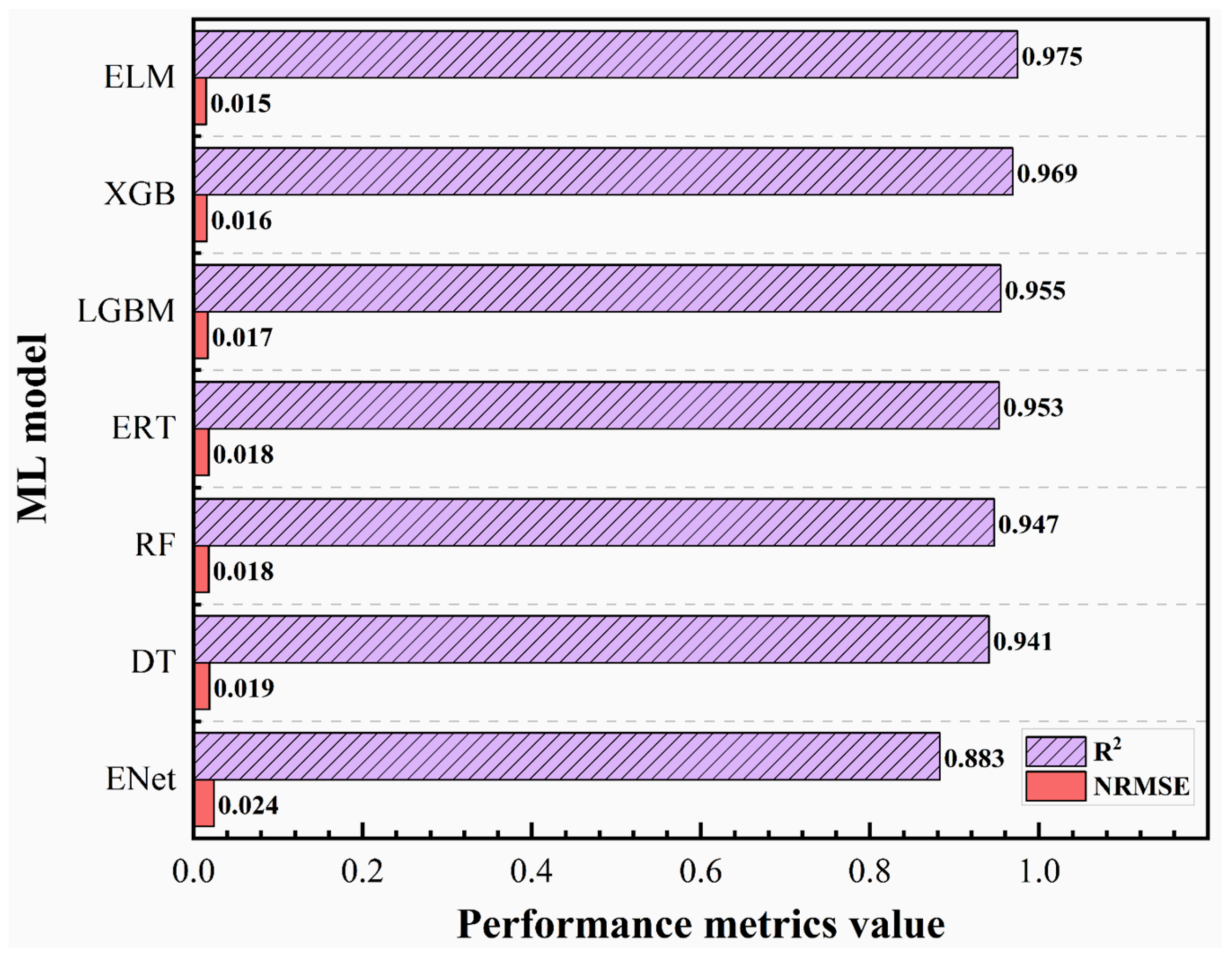
Comparison of the predictive performance of the candidate base learners and the final ELM.

**Figure 12:**
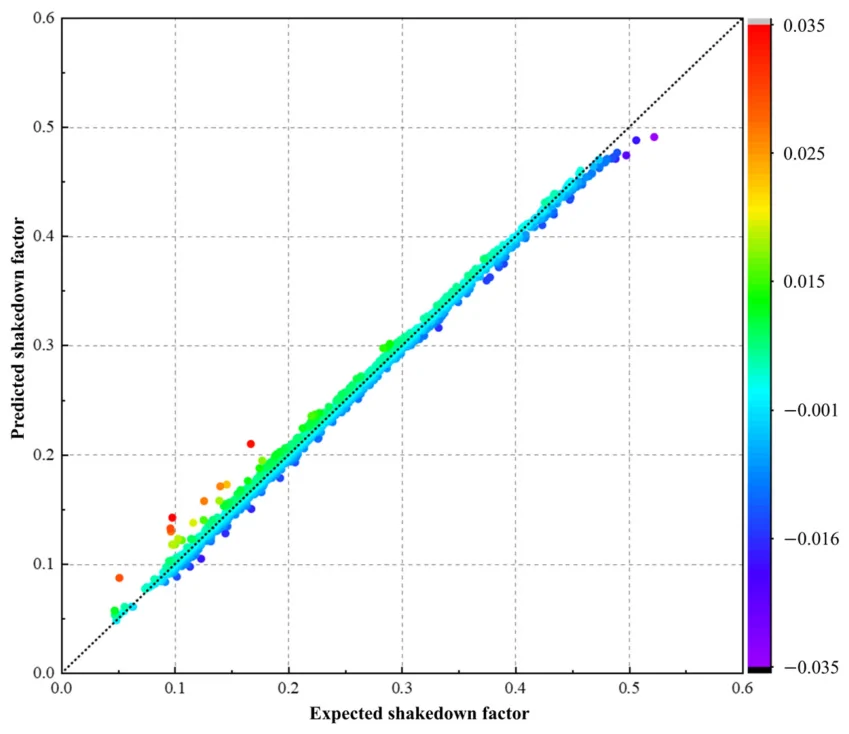
$\alpha^{SD}$ comparison between predicted and numerically computed values.

The final ELM is constructed by combining three optimized pipelines: MinMaxScaler–RF, RobustScaler–ENet, and MaxAbsScaler–LGBM. As shown in Figure 11, the final ELM provides the most favorable overall predictive performance among the evaluated models, demonstrating the benefit of combining learners with different regression characteristics. To assess its generalization capability and the potential for overfitting, the model is further evaluated using five repeated random holdout evaluations. The mean training $R^{2}$ and NRMSE are 0.9773±0.0001 and 0.0104±0.0002, respectively, whereas the corresponding testing values are 0.975±0.0003 and 0.015±0.0006. The resulting generalization gaps are approximately 0.0023 for $R^{2}$ and 0.0046 for NRMSE. The close agreement between the training and testing scores indicates that the ELM maintains high predictive accuracy for unseen samples and shows no marked evidence of overfitting. Figure 12 further demonstrates close agreement between the ELM predictions and the numerically computed shakedown factors across different geometric and loading configurations, supporting the robustness of the proposed model.

### 4.2. Factor Analyses

**Figure 13:**
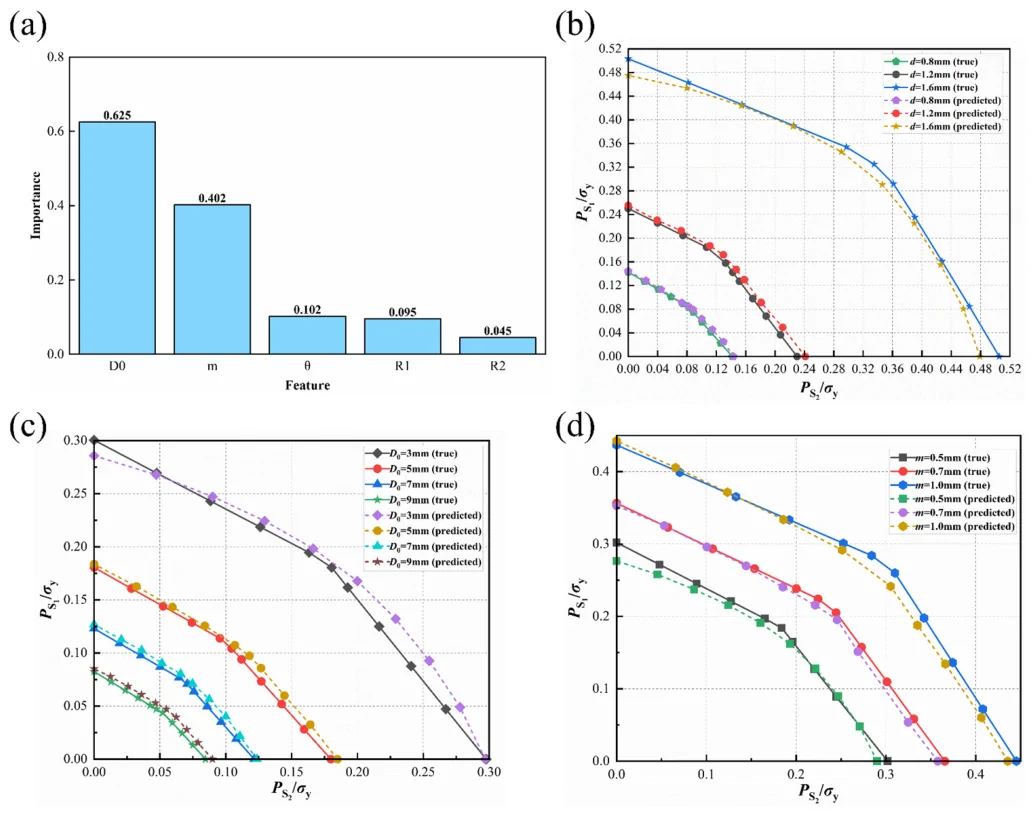
Parametric analyses of the BCC and PLA lattices: (**a**) feature importance of the geometric and loading variables for the PLA lattice; effects of (**b**) d, (**c**) $D_{0}$, and (**d**) m on the shakedown factor $\alpha^{SD}$. The reference domains were obtained from the FITD-based FE shakedown calculations, and the predicted domains were generated using the final voting-based ELM comprising the MinMaxScaler–RF, RobustScaler–ENet, and MaxAbsScaler–LGBM pipelines.

Using the proposed ELM, the feature importance of the PLA lattice is further analyzed, as shown in Figure 13a. The results show that $D_{0}$ and m are the dominant factors affecting $\alpha^{SD}$, with importance scores of 0.625 and 0.402, respectively, which are much higher than those of the other geometric variables. Although the influence of the biaxial loading parameter θ is weaker than that of $D_{0}$ and m, it remains non-negligible, particularly when the lattice geometry varies over a large design range.

A parametric study is then carried out to examine the effects of three representative design variables on $\alpha^{SD}$ for the BCC and PLA lattices. Figure 13b–d compares the numerically computed and predicted shakedown domains for variations in d for the BCC lattice and in $D_{0}$ and m for the PLA lattice. The true numerical reference values are obtained from the FITD-based FE shakedown calculations, whereas the predicted values are generated using the final voting-based ELM, which combines the MinMaxScaler–RF, RobustScaler–ENet, and MaxAbsScaler–LGBM pipelines. To quantify the agreement, the maximum pointwise absolute error is calculated as $e_{max}={max}_{i}|\alpha_{i,ELM}^{SD}-\alpha_{i,num}^{SD}|$ over all loading angles and parameter levels shown in each subfigure. As summarized in Table 6, the maximum errors associated with d, $D_{0}$, and m are 0.027, 0.014, and 0.027, respectively. These limited deviations confirm the predictive accuracy of the final ELM for reconstructing the elastic shakedown domains. The observed variation trends are also consistent with the feature-importance results in Figure 13a. Specifically, the shakedown domain increases markedly with increasing d for the BCC lattice and increasing $D_{0}$ for the PLA lattice, indicating positive correlations between these parameters and the shakedown capacity. In contrast, increasing m reduces the shakedown capacity of the PLA lattice and contracts the corresponding domain. These results provide useful guidance for fatigue-oriented ESC design at the early product-development stage.

## 5. Conclusions

This study develops a multiscale framework for evaluating and predicting the shakedown-based fatigue capacity of parameterized lattice structures for lightweight energy storage containers. The framework integrates an NCPBC formulation, FITD-based lower-bound shakedown analysis, and an ensemble-learning surrogate. The NCPBC formulation enables periodic constraints to be imposed on lattice RVEs with non-matching surface meshes, while the FITD scheme provides reliable stress evaluation for geometrically complex lattice members. Together, these developments allow the multiaxial fatigue capacity to be assessed without explicitly tracing the complete cyclic loading history.

The accuracy of the proposed framework is supported by benchmark comparisons, RVE-to-structure stress-field evaluations, experimental observations, and cyclic incremental analyses. The FITD-based shakedown solution shows a maximum relative error of 0.7% compared with published reference results. The trained ELM achieves an $R^{2}$ of 0.975 and an NRMSE of 0.015, demonstrating its ability to reproduce the numerically calculated shakedown factors. After offline training, the ELM predicts the capacity of new geometric and loading configurations without repeated FE analysis and GUROBI-based optimization, thereby improving the efficiency of design-space exploration.

The parametric results reveal distinct geometric effects for the two lattice configurations. Increasing the strut diameter d improves the shakedown capacity of the BCC lattice, whereas the PLA lattice is governed primarily by the center distance $D_{0}$ and edge width m. These findings establish quantitative relationships between mesoscale geometry and multiaxial shakedown capacity. Overall, the proposed framework provides a physics-based and computationally efficient approach for fatigue-oriented design and optimization of lattice-integrated lightweight energy storage container structures.

Future work will extend the proposed framework to less symmetric lattice configurations subjected to more general multiaxial loading conditions. Further experimental validation under representative road- and marine-transport load spectra will also be conducted to assess its applicability to practical energy storage container structures.

## Figures and Tables

**Table 1:** Material properties and design variables of the BCC lattice.

BCC Lattice	Design Variables	Resin Properties		
	*d* (mm)	*E* (GPa)	υ	$\sigma_{y}$ (MPa)
Initial value	1	2.62	0.4	80
Varying range	[0.8, 1.6]			

**Table 2:** Material properties and design variables of the PLA lattice.

PLA Lattice	Design Variables				SPA-H Properties		
	$R_{1}$ (mm)	$R_{2}$ (mm)	m (mm)	$D_{0}$ (mm)	*E* (GPa)	υ	$\sigma_{y}$ (MPa)
Initial value	4.025	1.875	0.5	6.25	200	0.3	355
Varying range	[2, 6]	[1, 3]	[0.5, 1.0]	[3, 9]			

**Table 3:** Material properties of the plate with a hole model.

*E* (GPa)	υ	$\sigma_{y}$ (MPa)
200	0.3	200

**Table 4:** Mesh-convergence results for the plate-with-a-central-hole model discretized using different element sizes.

Mechanical Response Type	Mesh Size				
	2 mm	1.5 mm	1 mm	0.7 mm	0.5 mm
Equivalent stress-Max. (Mpa)	155.8	163.55	166.26	166.85	166.96
Equivalent stress-Ave. (Mpa)	55.13	54.27	53.73	53.38	53.21
Element number	6307	15,542	46,802	132,787	201,938

**Table 5:** Shakedown result comparisons with research.

Authors and Reference	Shakedown Loading Conditions	
	$P_{2}=0$	$P_{1}=P_{2}$
Simon et al. [34]	0.627	0.458
Garcea et al. [35]	0.604	0.438
Tran et al. [36]	0.601	0.434
G. Zhang [37] ^a^	0.596	0.431
Author’s result (FITD)	0.595	0.428

^a.^ Comparison reference result of FITD-based shakedown evaluation.

**Table 6:** Maximum absolute errors between the numerically computed and ELM-predicted shakedown factors for the parametric cases shown in Figure 13.

Lattice	Design Variable	Parameter Levels Shown in Figure 13	Maximum Absolute Error in $\alpha^{SD}$
BCC	d	0.8, 1.2, and 1.6 mm	0.027
PLA	$D_{0}$	3, 5, 7, and 9 mm	0.014
PLA	m	0.5, 0.7, and 1.0 mm	0.027

Note: The maximum absolute error was calculated over all loading angles and parameter levels presented in the corresponding subfigure.

## Data Availability

The original contributions presented in this study are included in the article. Further inquiries can be directed to the corresponding author.

## Abbreviations and Symbols

Main abbreviations		
Abbreviation	Definition	
BCC	Body-centered cubic	
BCC+CP	Body-centered cubic with cubic-primitive	
BESS	Battery energy storage system	
DT	Decision tree	
ELM	Ensemble-learning model	
ENet	Elastic Net	
ERT	Extremely randomized trees	
ESC	Energy storage container	
FE	Finite element	
FITD	Full-integration tetrahedral discretization	
GBDT	Gradient-boosted decision trees	
GCS	Global coordinate system	
LGBM	Light Gradient Boosting Machine	
NCPBC	Nodal-coupling periodic boundary condition	
NCS	Natural coordinate system	
NRMSE	Normalized root mean squared error	
PBC	Periodic boundary condition	
PLA	Peanut-like auxetic	
RF	Random forest	
RMSE	Root mean squared error	
RVE	Representative volume element	
SEM	Scanning electron microscopy	
SPA-H	Weathering structural steel	
XGB	Extreme Gradient Boosting	
Main symbols		
Symbol	Definition	Unit
$A_{s}$	Cross-sectional area of the PLA lattice	mm^2^
B	Element strain-displacement (geometric) matrix	—
$B^{*}$	Geometric matrix in the natural coordinate system	—
$L_{0}$, $D_{0}$, $T_{0}$	Length, center-distance, and thickness of the PLA lattice	mm
d	Strut diameter of the BCC lattice	mm
E	Young’s modulus	GPa
G	Geometric spacing parameter of the PLA lattice	mm
J	Jacobian matrix	—
$L_{1}$, $D_{1}$, $T_{1}$	Length, center-distance, and thickness of the BCC lattice	mm
m	Edge-width parameter of the PLA lattice	mm
[N]	Finite-element shape-function matrix	—
NG	Total number of Gauss points	—
NE	Total number of finite elements	—
NGE	Number of Gauss points per element	—
$P_{1}$, $P_{2}$, $P_{S_{1}}$, $P_{S_{2}}$	Independent load components	MPa
$R_{1}$	Inner-radius parameter of the PLA lattice	mm
$R_{2}$	Outer-radius parameter of the PLA lattice	mm
$R^{2}$	Coefficient of determination	—
$S_{i}$, $S_{i}^{\prime }$	Corresponding periodic surfaces of the lattice RVE	—
$E_{BCC}$	Equivalent Young’s modulus of the BCC lattice	MPa
t	Time or loading-history parameter	s
$u_{x}$, $u_{y}$, $u_{z}$	Nodal displacement vector	mm
w	Gauss integration weight	—
$\alpha^{SD}$	Shakedown load factor	—
ε	Local strain tensor	—
E	Global strain tensor	—
θ	Biaxial load-proportion parameter	—
$\mu_{1}$	Load multiplier associated with the i-th load basis	—
$\mu_{1}^{-}$, $\mu_{1}^{+}$	Lower and upper bounds of the i-th load multiplier	—
υ	Poisson’s ratio	—
$\bar{\rho }$	Time-independent self-equilibrated residual stress field	MPa
$\sigma^{E}$	Elastic stress field generated by the prescribed load	MPa
Σ	Macroscopic stress tensor	MPa
$\sigma_{y}$	Material yield strength	MPa
D	Elastic tensor	—
[C]	Assembled equilibrium matrix	—
L	Admissible multidimensional load domain	—
〈·〉	Volume average operator	—
F(σ)	Yield function	—
${\left\{\delta \right\}}^{e}$	Element nodal-displacement vector	mm
∂	Differential-operator matrix	—
$\tilde{u}$, $\tilde{\varepsilon }$	Periodic displacement and strain fluctuation	mm, —
η	Solid volume fraction of the BCC lattice	%
$e_{max}$	Maximum pointwise absolute error	—
$\alpha_{i,ELM}^{SD}$, $\alpha_{i,num}^{SD}$	ELM-predicted and numerically computed shakedown factors	—
